# Exosomes in Cancer Biology: Emerging Biomarkers and Therapeutic Targets

**DOI:** 10.7150/jca.132498

**Published:** 2026-06-10

**Authors:** Marriam Ahmed, Chieh-Wei Chang, Sundas Ijaz, Abdul Qadeer, Khalid J. Alzahrani, Khalaf F. Alsharif, Fuad M. Alzahrani, Abdulwahab Abuderman, Chien-Chin Chen, Shahid Hussain

**Affiliations:** 1 Department of Biotechnology, Kohsar University, Murree, Pakistan.; 2 Division of General Surgery, Department of Surgery, Ditmanson Medical Foundation Chia-Yi Christian Hospital, Chiayi 60002, Taiwan.; 3 School of Medical Sciences, Shandong Xiehe University, Jinan 250109, China.; 4 Department of Clinical Laboratories Sciences, College of Applied Medical Sciences, Taif University, P.O. Box 11099, Taif 21944, Saudi Arabia.; 5 Department of Pathology, Ditmanson Medical Foundation Chia-Yi Christian Hospital, Chiayi 600, Taiwan.; 6 Department of Cosmetic Science, Chia Nan University of Pharmacy and Science, Tainan 717, Taiwan.; 7 Doctoral Program in Translational Medicine, National Chung Hsing University, Taichung 402, Taiwan.; 8 Department of Biotechnology and Bioindustry Sciences, College of Bioscience and Biotechnology, National Cheng Kung University, Tainan 701, Taiwan.; 9 Basic Medical Sciences Department, College of Medicine, Prince Sattam Bin Abdulaziz University, Al-Kharj, Saudi Arabia.

**Keywords:** microvesicles, biomarker, angiogenesis, cell-to-cell communication, annexins

## Abstract

Exosomes are small extracellular vesicles (EVs) that play an important role in intercellular communication among multiple cell types. In recent years, they have emerged as a novel and promising class of cancer biomarkers, offering significant potential to increase diagnostic and therapeutic strategies. These bilayer nano-vesicles are actively secreted by living cells into various biological fluids and carry a diverse cargo of proteins, nucleic acids, and other biomolecules that reflect the physiological and pathological state of their cells of origin. The molecular composition of exosomes mirrors the dynamic processes and unique cargo of molecular and genetic data, reflecting the complex activities within cancer cells, making them a promising alternative for cancer detection and treatment monitoring. Although the mechanisms underlying exosome biogenesis, secretion, and cargo selection in cancer remain incompletely understood, growing evidence highlights their importance in tumor progression and therapeutic response. Exosomal proteins have gained considerable attention as potential therapeutic targets. These proteins can regulate immune responses, reshape the tumor microenvironment, and influence cancer cell proliferation and survival. Consequently, targeting exosome-associated proteins represents a promising strategy for developing innovative anticancer therapies. Advances in exosomal protein analysis have provided a promising approach for unraveling the complex molecular networks underlying cancer biology. A wide range of analytical techniques is available to identify and quantify exosomal proteins, enabling characterization of cancer-specific molecular signatures. As an expanding field in cancer research, exosomes have the potential to revolutionize both therapies and diagnostics. By deciphering the diverse molecular and functional cargos of exosomes, exosomes offer new insights that may lead to more precise, effective, and personalized approaches to cancer management.

## Introduction

Extracellular vesicles (EVs) are lipid bilayer-enclosed particles secreted by nearly all cell types and play a crucial role in intercellular communication 1. These vesicles include exosomes, microvesicles (MVs), ectosomes, and apoptotic bodies. They serve as vital carriers of a diverse range of biomolecules 2, 3. Among them, exosomes have attracted particular attention due to their involvement in both physiological and pathological processes, especially cancer. Exosomes are typically 30-150 nm in diameter and originate from the endosomal pathway 4. Their biogenesis is a complex process that begins with inward budding of endosomal membranes to form multivesicular bodies (MVBs). These MVBs contain intraluminal vesicles (ILVs) and can follow one of several fates. They can fuse with lysosomes for degradation, remain distinct within the cytoplasm, or fuse with the plasma membrane to release their ILVs into the extracellular environment as exosomes 5. This intricate process of endocytosis is followed by exocytosis, which is regulated by various proteins. These proteins include GTPases such as Rab, components of the ESCRT-III complex, and Alix, which is involved in exosome synthesis and transport 6.

Exosomes were first identified in 1983 by Rose Johnstone during reticulocyte maturation. They were initially dismissed as a mechanism by which cells dispose of waste products 7. However, this view changed in 1996 when B-cell-derived exosomes were shown to possess antigen-presenting properties capable of activating T-cell responses 8. Since then, exosomes have been recognized as important mediators of intercellular signaling, capable of transferring bioactive cargo that modulates recipient cell behavior 9. In cancer biology, exosomes contribute to tumor progression, immune modulation, and therapeutic resistance. Their molecular composition often reflects their cell of origin, highlighting their potential as minimally invasive diagnostic biomarkers and therapeutic delivery vehicles 10. Emerging evidence also suggests that regulatory pathways, including those associated with peptidyl arginine deiminase (PAD), may influence exosome-mediated tumor dynamics 11. Therefore, a thorough understanding of exosomal proteins and their functions is crucial for advancing cancer diagnostics and developing innovative therapeutic strategies. This review will delve into the multifaceted roles of exosomes in cancer, focusing on techniques for analyzing their protein content and the specific functions of these proteins in the disease.

## Literature Search Strategy

An extensive literature search was conducted to identify relevant literature on the role of exosomes in cancer progression, diagnosis, and treatment. The search of electronic databases such as PubMed, Scopus, Web of Science, and Google Scholar was conducted systematically to identify articles published between January 2013 and December 2025. A combination of keywords related to exosomes and cancer was used as the search strategy along with the Boolean operators (AND, OR). The key search terms were “exosomes,” “extracellular vesicles,” “small extracellular vesicles,” “cancer,” “tumor,” “neoplasm,” “metastasis,” “angiogenesis,” “tumor microenvironment,” “biomarkers,” “drug delivery,” and “therapeutic applications”. Peer-reviewed articles were considered only in English. Conference abstracts, non-English publications, and irrelevant studies to cancer biology were excluded.

## Techniques for Analyzing Exosomal Protein Contents

The protein composition of exosomes can be examined using various techniques, including mass spectrometry, Western blotting, enzyme-linked immunosorbent assay (ELISA), and fluorescence-activated cell sorting (FACS). Researchers have extensively characterized the protein cargo of cancer-derived exosomes using mass spectrometry (MS) based approaches. Advancements in mass spectrometry-based proteomics now enable comprehensive, quantitative profiling of global proteomes from cells, tissues, and biological fluids. When integrated with complementary omics datasets, proteomic analysis provides deeper insight into disease-associated molecular mechanisms. This facilitates the discovery of novel diagnostic biomarkers and the identification of potential therapeutic targets. Recent studies using MS-based proteomic analysis of EVs secreted by cancer cells have revealed key biogenesis pathways, enhancing our understanding of carcinogenesis and tumor progression. In parallel, Western blotting has played an important role in exosome research by enabling the detection and quantification of specific exosomal proteins. This technique has been instrumental in identifying proteins associated with cancer dissemination and prognosis 12.

The ELISA technique is particularly well-suited for analyzing vesicles from both human body fluids and cell culture supernatants. It selects only the relevant number of vesicles and assumes that antigens can only be produced by vesicles ranging from the nanoscale to the microscale. Exosome research has benefited significantly from ELISA, which enables scientists to identify and quantify specific exosomal proteins secreted by cancer cells. The method has been used to pinpoint and measure exosomal proteins implicated in the spread and progression of cancer 13. Numerous optical and non-optical methods have been developed to assess the size, size distribution, shape, quantity, and biochemical content of isolated exosomes, thereby evaluating their quality. 14. Since preparative ultracentrifugation is used to separate minute bioparticles such as bacteria, viruses, extracellular vesicles, and subcellular organelles, it is crucial to the extraction of exosomes. As one of the most widely used and documented methods for isolating exosomes, ultracentrifugation-based exosome isolation is regarded as the gold standard. 56% of all exosome isolation methods used by users in exosome research are thought to be ultracentrifugation 15. During exosome isolation, the supernatant is aspirated between runs. Depending on the centrifugal force used, either the pellet or the supernatant is resuspended in a suitable medium, such as phosphate-buffered saline (PBS), and subjected to further centrifugation at increasing centrifugal force. The separated exosomes are then resuspended and stored at -80°C until further examination. The pelleting approach and the simple ultracentrifugation method are other names for this exosome isolation technique 16. Ultrafiltration is a widely used size-based exosome isolation method. The principles of ultrafiltration are identical to those of traditional membrane filtration, where the size or molecular weight of suspended particles or polymers determines their separation. Consequently, membrane filters with specified molecular weight or size-exclusion criteria can be used to separate exosomes by size. 17. A method for isolating and purifying biological particles based on antigen-antibody specificity is called immunoaffinity. Exosome membranes are abundant in proteins and receptors, containing both unique and widely distributed common proteins. These common proteins, such as tetraspanins and annexins, can bind specific antibodies to isolate exosomes in a targeted manner 18.

In scientific studies, exosomes are retrieved through repeated centrifugation. Low-frequency centrifugation is used to separate undesired cells and cellular debris at least 2 or 3 times. Using specific protein membranes, immune isolation can isolate purified subpopulations of EVs. Details of the various diagnostic techniques used for exosomal protein identification are summarized in Table 1.

## Therapeutic Targeting Strategies

Because of their functional characteristics, exosomes have attracted considerable interest recently as a potential delivery vehicle 24. These vesicles offer several benefits, including good biocompatibility, low immunogenicity, and target-tissue selectivity 25. Because of their lipid bilayer structure, the cargo inside exosomes is stable and cannot be broken down by enzymes or other substances. Exosomes are also ideal delivery vehicles for small-molecule medications due to their exceptional properties. For example, they can precisely deliver natural cargoes such as proteins, DNA, siRNA, and miRNA to their intended destinations 26. Unaltered blood exosomes were shown by Qu *et al*. to efficiently target the brain and release dopamine as a Parkinson's disease treatment. 8. Exosomes containing small-molecule medications have demonstrated encouraging therapeutic outcomes, and this delivery method can reduce side effects while maintaining the medications' pharmacological effects 27. As the name implies, the incubation approach involves co-incubating exosomes with small compounds for a predetermined period. This procedure often enables the loading of small compounds into exosomes. This method is easy to use and doesn't compromise the integrity of the exosomes. The loading efficiency with this method depends on the polarity of the small molecule. Catalase and other low- to medium-molecular-weight lipophilic small molecules are more readily loaded into exosomes via incubation 28. By electroporating DOX into exosomes derived from lens epithelial cells, Zhu *et al*. were able to fix the loaded exosomes to the surface of an artificial lens, resulting in efficient uptake by lens epithelial cells and notable anti-proliferative effects 29. Compared with other approaches, electroporation is a straightforward and effective method for loading pharmaceuticals into exosomes while preserving the original drug characteristics. However, electroporation conditions affect loading efficiency. Lennaárd *et al*. showed that loading efficiency could be greatly increased under suitable electroporation conditions by systematically evaluating factors such as the total number of EVs, drug-to-vesicle ratio, electroporation buffer solution, pulse capacitance, and field strength 30. Sonication is a technique that enables the loading of small-molecule medications into exosomes by applying mechanical shear force with an ultrasound probe to disrupt the exosomal membrane 31.

## Exosomes in Cancer Progression

All prokaryotic and eukaryotic cells can release EVs through evolutionarily conserved mechanisms 32. Among these, exosomes have emerged as key regulators of cancer progression, contributing to tumor growth and immune evasion. Exosomes can function as carriers of bioactive molecules, including proteins, nucleic acids, and lipids, which are transferred to recipient cells, thereby altering their phenotype and behavior. Through this intracellular communication, exosomes can reshape gene expression profiles, alter the tumor microenvironment, and modulate signaling pathways, thereby accelerating cancer growth. Cancer cells secrete exosomes in a dysregulated manner, and these vesicles facilitate metastatic dissemination via paracrine and systemic signaling. Importantly, exosomal cargo varies by cancer type, conferring disease-specific functional properties that enable the transfer of oncogenic signals from primary tumors to distinct sites. Exosomes play a crucial role in the spread of metastatic lesions and, in rare cases, can even promote malignancy, for instance, when cancer cells release exosomes that contain the machinery for metastasis, or when exosomes from the primary lesion travel through the bloodstream to a distant organ. As was previously indicated, cells that migrate from the initial lesion and pass through blood arteries to reach a distant organ do so without being attacked by immune cells 33.

### Exosomal Genes and miRNAs

According to a 2014 study by Zhou *et al*., miR-105 is found in exosomes originating from metastatic breast cancer tissue. By acting on vascular endothelial cells, these exosomes suppress the expression of the adhesion protein ZO-1, ultimately promoting cancer cell migration out of blood vessels 34, 35. Through direct effects on tumor and endothelial cells and indirect modulation via exosomal communication, microRNA-155 (miR-155) plays a crucial role in controlling angiogenesis. Exosomal miR-155 promotes vascularization in several cancers by enhancing intercellular communication. Using antagomirs to suppress miR-155, exosome-mediated delivery methods, and mechanisms such as JAK2/STAT3 and TGF-β/SMAD2 are examples of developing therapeutic approaches. One promising strategy to prevent tumor angiogenesis and enhance the effectiveness of cancer treatment is to target miR-155 36.

By transferring bioactive molecules such as proteins, lipids, and miRNAs that inhibit cell death pathways and promote survival, exosomes function as powerful anti-apoptotic agents in cancer 37. In tumor-derived exosomes, miR-21, miR-221, miR-222, and miR-23a are frequently expressed and inhibit pro-apoptotic genes such as PTEN, PDCD4, and BIM. This activates the PI3K/AKT and MAPK/ERK signaling cascades, which, in turn, increase the expression of anti-apoptotic proteins such as Bcl-2 and survivin 38.

**Figure 1:**
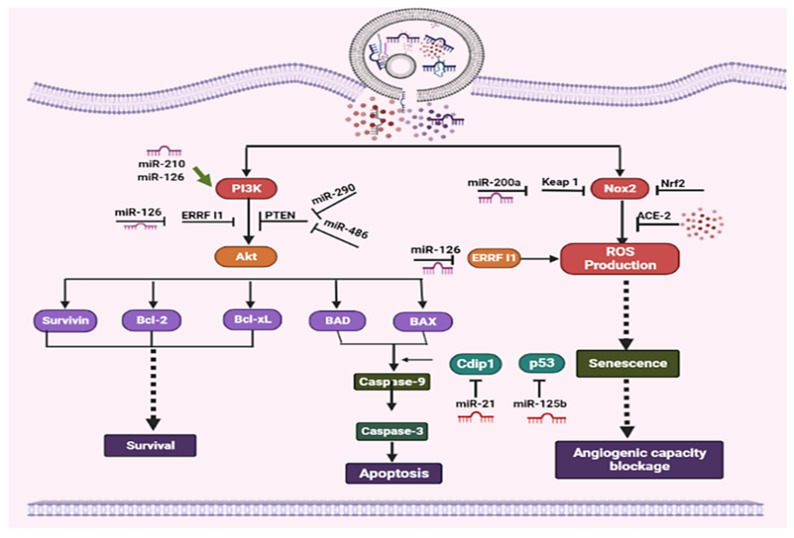
Anti-apoptotic activity of Exosomes: By boosting anti-apoptotic proteins, inhibiting pro-apoptotic pathways, and activating PI3K/Akt signaling, exosomes increase endothelium survival.

Exosomes are essential for regulating angiogenesis, apoptosis, and cell survival via multiple signaling pathways and microRNA-mediated mechanisms. A crucial survival pathway is the PI3K/Akt pathway, in which PI3K activates Akt to promote the expression of anti-apoptotic proteins such as Bcl-2, Bcl-xL, and Survivin, while suppressing pro-apoptotic proteins such as BAD and BAX. PTEN and ERRF1 function as negative regulators of this pathway, along with microRNAs such as miR-126, miR-210, miR-290, and miR-486. Apoptosis is controlled through the intrinsic pathway involving Caspase-9 and Caspase-3, with modulators such as Cdip1 and the tumor suppressor p53, which can be influenced by miR-21 and miR-125b. Reactive oxygen species (ROS) production, driven by Nox2 and modulated by the Keap1/Nrf2 axis, induces senescence and can block angiogenic capacity; microRNAs such as miR-200a and miR-126 further regulate oxidative stress and ERRF1 activity. Tumor-related surface proteins, such as ACE-2 and E-cadherin, carried by exosomes can influence microenvironmental responses, including angiogenesis and metastatic potential. Overall, exosomes integrate these complex pathways to balance survival, apoptosis, and senescence, with microRNAs acting as crucial modulators of gene expression in these processes 39. The functional role of exosomes is summarized in Figure 1.

### Importance of Exosomal Protein Content

In the realm of cancer studies, there is growing interest in investigating exosomes and their protein composition 40. These minuscule vesicles, which transport various substances such as proteins, nucleic acids, and lipids, are crucial for intercellular communication. Both healthy and cancerous cells release them, and the physiological and pathological states of the parent cells are reflected in their contents 41. Exosomes have reportedly been shown to direct distant organs towards a premetastatic niche, a favorable microenvironment that promotes the survival and growth of tumor cells 42. Exosomes can be subtyped based on surface markers without compromising their structural integrity, since exosomal proteins are either embedded in the membrane or confined within the lumen. Specifically, proteins such as CD63, TSG101, and Alix have been identified as biomarkers for exosomes produced by various cell types. In contrast, several other proteins, including Calnexin, serve as barriers to exosome detection. Exosomes released by cells undergoing pathological processes have unique compositions that serve as indicators of those cells' statuses. Exosomes' surface proteins, such as EGFR, EphA2, and EpCAM, are increasingly used to differentiate tumor-derived (TD) exosomes from non-tumor exosomes. Exosomes carrying E-cadherin, a factor, and angiogenin can be produced in large quantities by metastatic ovarian cancer (OC) cells 43.

### Common Exosome Proteins in Cancer

#### Heat Shock Proteins

The molecular chaperones known as heat shock proteins (HSPs) are vital for maintaining proteostasis and cellular processes, stabilizing protein structures, and aiding in the degradation or repair of damaged proteins. Numerous studies have shown that HSPs are linked explicitly to the development and spread of cancer and are elevated in these cells 44. HSPs have been found in exosomes produced by cancer cells. This has led scientists to investigate the potential role of exosomal HSPs in the emergence of cancer and in resistance to cancer therapies. Numerous studies on cancer biology have identified countless potential therapeutic targets. A molecular chaperone is one of these. It belongs to the group of proteins called HSPs. HSPs are found in all mammalian cells and are essential for the proper folding of newly synthesized proteins and for the refolding of denatured proteins in response to a wide range of internal and external stimuli. HSPs play a crucial role in maintaining protein quality. These circumstances include abrupt temperature fluctuations, exposure to high amounts of reactive oxygen species (ROS), and severe cellular damage that affects the structure and longevity of proteins, as reported by 45). In their hypothesis that malignant cells are "addicted to chaperones". Researchers studying the function of HSPs in cancer emphasized the importance of three well-studied HSPs, including HSP70, HSP27, and HSP90, in the development of tumor therapies 46. Some HSPs are also associated with chemotherapy. Consequently, targeting Exosome HSP90 may be a promising approach for developing novel cancer treatments. Heat shock proteins play a vital role in chemotherapy resistance (Table 2).

#### Integrin

Integrins, which are critical surface adhesion receptors that mediate interactions between cells and the extracellular matrix (ECM), are essential for cell migration and tissue homeostasis. Initial tumor formation, growth, and metastasis are facilitated by aberrant integrin activation. Several lines of evidence have recently shown that integrins play diverse roles in tumorigenesis and are highly expressed across a wide range of cancer types 56. Exosomal Integrins are involved in several processes that promote cancer growth, such as enhancing cell attachment and migration and directing cancer exosomes to specific metastatic sites. The balance between intracellular activators, such as talin and kindlin, and inactivators, such as the Shank-related RH domain interactor (SHARPIN) and integrin cytoplasmic domain-linked protein 1 (ICAP-1), controls activation-dependent conformational modifications. These regulate integrin function within cells. Exosomal integrins have attracted considerable attention lately, and it is evident that they play a role in establishing premetastatic niches, regulating the tissue distribution of exosomes, facilitating exosomal internalization by target cells, and mediating the transport of membrane proteins and related kinases to target cells via exosomes. There is increasing evidence that tumor and immune cell exosomes can alter endothelial properties (migration, proliferation) and gene expression 57, with vesicle-bound integrins promoting these effects. We also discuss how tumor cells and their exosomes pleiotropically alter endothelial activities within the tumor microenvironment, as endothelial metabolism is currently believed to be crucial for tumor angiogenesis 58.

**Figure 2:**
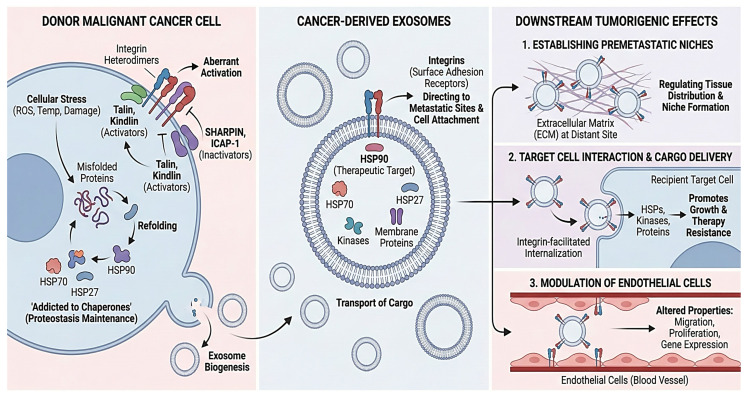
Integrins and chaperones in exosome-mediated metastasis. Stress causes cancer cells to upregulate HSPs (HSP90, HSP70, and HSP27) and control integrins before releasing exosomes. Chaperones, kinases, and integrins that direct organ-specific targeting are carried by exosomes. By creating premetastatic habitats, improving recipient cell survival and therapy resistance, and altering endothelial cell function, these vesicles facilitate metastasis.

Cell differentiation and spread 59, organ development and tissue regeneration mechano-transduction 60, and inflammation are mediated by integrins. Integrins are heterodimeric membrane proteins made up of α and β subunits. They are essential components of integrin adhesion complexes and play a key role in transmitting bidirectional transmembrane signals at focal adhesions 60. Every single integrin heterodimer in mammals consists of an α-subunit and a β-subunit in a noncovalent complex. Together, the 18 α- and 8 β-subunits form 24 functionally unique heterodimeric receptors that contribute to a variety of activities, including signal transduction, cell migration, and cell adhesion 61. One of the most notable properties of integrins is their ability to transmit directional transmembrane signals through conformational changes 62. When additional receptors, such as growth factors and chemokine receptors, are activated, intracellular signals are released 63. According to another study, integrins are heterodimeric membrane proteins composed of α and β subunits. They are a significant component of integrin-adhesion complexes in focal adhesions and are essential for mediating bidirectional transmembrane signaling 60. The integrin subunit is a type I transmembrane glycoprotein distinguished by its short cytoplasmic (carboxyl-terminal) tail, single transmembrane helix, and large extracellular domain. Both the α- and β-subunits have carboxyl termini that extend across the plasma membrane, both extracellularly and intracellularly. The amino-terminal regions of the α- and β-subunits combine to form integrin heterodimers, which are active receptors that mediate cell-extracellular matrix interactions 61. The detailed overview is presented in Figure 2.

#### Annexins

**Figure 3:**
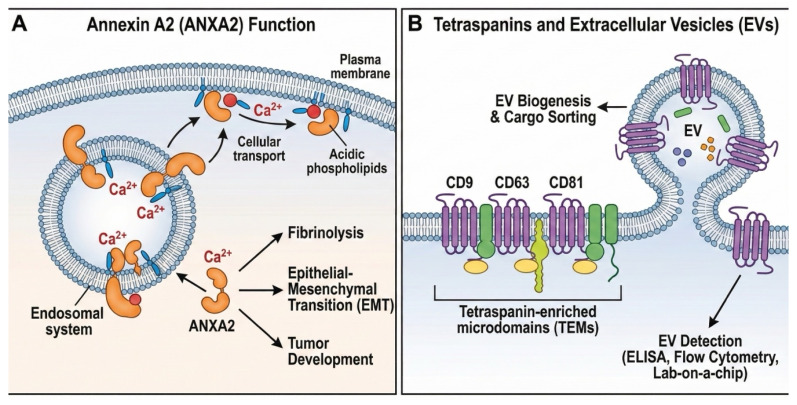
Tetraspanins and Annexin A2 in extracellular vesicle biology. (A) ANXA2 controls transport, fibrinolysis, EMT, and tumor growth by mediating Ca2+-dependent membrane-cytoskeleton interactions. (B) Tetraspanins (CD9, CD63, and CD81) are important indicators for EV detection and arrange membrane microdomains necessary for EV production and cargo sorting.

At high Ca2+ concentrations, cytosolic proteins called annexins, which have conserved three-dimensional structures, bind acidic phospholipids in cellular membranes. By doing this, they organize membrane lipids and function as Ca2+-regulated membrane-binding modules, promoting cellular membrane transport while simultaneously exhibiting extracellular activity. The term "Annexin" derives from the Greek "annex," meaning "bring/hold together," and was chosen to signify the primary trait that all, or almost all, annexins share 64. At least 20 family members have been identified thus far 9. One of the better-characterized Annexins is Annexin A2 (ANXA2), also known as Annexin II. The two primary structural domains of ANXA2 are the 33-kDa C-terminal conserved core domain and the membrane- and Ca2+-binding sites, respectively 65. AnxA2, a Ca2+-dependent phospholipid-binding protein, connects the endosomal and plasma membrane systems. According to another study, ANXA2 is a member of the Annexin A family and is involved in fibrinolysis, the epithelial-mesenchymal transition, and various other physiological processes. In earlier research, annexin A2 has been widely linked to tumor development and genesis 66, as shown in Figure 3A.

#### Tetraspanin

Tetraspanins constitute a major protein superfamily that organizes membrane microdomains known as tetraspanin-enriched microdomains (TEMs) and are highly abundant in EVs. These proteins interact with a range of transmembrane and cytosolic signaling molecules, assembling them into functional clusters. Among tetraspanins, CD9, CD63, and CD81 are the most commonly reported EV-associated markers in the literature and have been extensively used for EV detection and isolation in techniques such as ELISA, flow cytometry, and lab-on-a-chip assays 67. These markers are frequently used to capture the total EV population. Importantly, growing evidence indicates that CD9, CD63, and CD81 play an active role in EV biogenesis or cargo sorting, underscoring their essential involvement in the EV secretory pathway 68, as shown in Figure 3B.

### Tumor Antigens

T cells produce exosomes that reflect their functional activities, including cytotoxic responses, modulation of B-cell antibody production, antigen recognition, and cytokine secretion, which together shape the immune microenvironment via paracrine and autocrine signaling 69. These exosomes transfer regulatory molecules, such as miRNAs, surface receptors, and signaling proteins, to neighboring cells or back to the T cells themselves, modulating transcription, cytokine secretion, proliferation, and differentiation to fine-tune immune responses. In cancer, T-cell exosomes can carry tumor-associated antigens (TAAs) and immune-stimulating molecules, enhancing antigen presentation, cytotoxic activity, and immune surveillance, while tumors may alter exosome content or abundance to suppress immunity and facilitate evasion. Consequently, T-cell exosome profiles can indicate immune suppression within the tumor microenvironment. Given their dual role in modulating anti-tumor immunity and reflecting tumor immunological status, T-cell-derived exosomes are being investigated as potential diagnostic biomarkers and therapeutic agents, including strategies to engineer exosomes with immunostimulatory molecules or tumor antigens to boost anti-tumor responses, as well as profiling patient-derived exosomes to monitor tumor progression, immune activity, and therapy efficacy, offering a non-invasive tool for precision oncology 35.

### Exosomal Proteins and Their Functional Roles in Cancer

Exosomes can be subtyped based on surface markers without compromising their structural integrity since exosomal proteins are either embedded on their surface or encapsulated within the exosome lumen 70. Exosomes include a range of different proteins. It consists of proteins made by cells as well as self-proteins. Based on the following properties, exosomal proteins from cancerous cells are emerging as potential biomarkers for cancer surveillance and evaluation of treatment effectiveness. Exosomes can be used to test for cancer, as they contain cancer-related lipids, proteins, RNA, and DNA.

#### Regulation of Cell Growth and Survival

Exosomes contain a range of biomolecules, including proteins, lipids, and nucleic acids. Heat Shock Proteins (HSPs) are among the proteins found in exosomes. They have been identified as having a part in controlling cell development and survival. During autophagy, Hsp90 is crucial. By suppressing mTOR, an Hsp90 inhibitor promotes autophagy 71. Starvation triggers autophagy, a process that uses self-digestion as an alternative energy source. Thus, autophagy acts as a short-term survival strategy. The activation of tumor cell death also involves autophagy, and excessive autophagy can lead to autophagic cell death in tumors 72. Overall, the discovery of these exosomal proteins and their roles in promoting cancer cell survival highlights the complex nature of cancer and underscores the importance of understanding its molecular mechanisms for developing effective treatments.

#### Facilitation of Metastasis

Exosomes are tiny vesicles that cells produce and contain various proteins and chemicals. Exosomes can promote tumor invasion and spread by disrupting angiogenesis and other critical cancer-related processes 38. Similarly, another study found that the tetraspanin CD151 is implicated in the motility, spread, angiogenesis, and proliferation of cancer cells. The molecular pathways underlying cancer development have been clarified by the discovery of these exosomal proteins and their roles in promoting metastasis. The development of treatments aimed at preventing or curing metastasis, a significant issue in cancer treatment, may identify new targets as a result of a better understanding of these pathways 73, 74.

#### Immune Evasion

**Figure 4:**
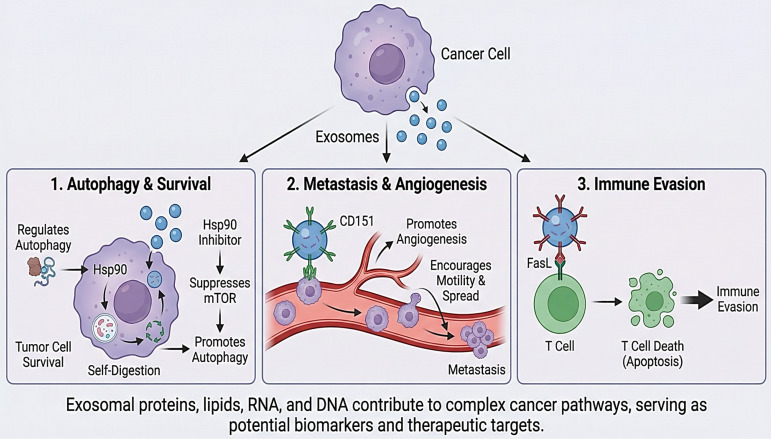
Cancer progression mediated by exosomes. Proteins, lipids, and nucleic acids carried by tumor-derived exosomes highlight their functions as biomarkers and therapeutic targets by promoting (1) autophagy and survival via Hsp90-mTOR signaling, (2) metastasis and angiogenesis through markers like CD151, and (3) immune evasion via FasL-induced T cell apoptosis.

Recent studies have demonstrated that exosomal proteins, such as tumor antigens, may significantly contribute to cancer cells' immune evasion. To accomplish immunological escape, exosomes can impair the immune response and cause T cell death. Malignant tumors have higher levels of the FasL transmembrane protein, a member of the TNF protein family. Exosomes generated from tumors overexpress FasL 75. Additionally, recent studies have shown that tumor cells can either naturally produce immune-modulating exosomes or be prompted to do so, and that the components of these exosomes contribute to cancer growth. Exosomes may facilitate metastatic colonization of secondary organs. They deduced that exosomal Integrins govern exosome uptake, which, in turn, causes resident cells at tropic metastatic sites to activate Src and upregulate S100 genes. These pro-migratory and inflammatory signals have extracellular immunological effects, such as mobilizing myeloid cells from bone marrow, thereby worsening inflammation 76. Overall, the discovery of exosomal proteins and their roles in immune evasion underscores the need to develop innovative strategies for cancer treatment, as shown in **Figure 4**.

#### Angiogenesis

Recent investigations have suggested that exosomal proteins are involved in angiogenesis, the process by which blood vessels develop from preexisting vessels. Angiogenesis is the process through which new capillaries form from the existing vascular system. This process is regulated by a variety of growth factors and signaling pathways, which also depend on the right balance of pro- and anti-angiogenic molecules. Recent investigations have shown that cell-derived microparticles (Microvesicles and exosomes) can also influence angiogenesis 77. Exosomes produced by cancer cells, which represent the parent cells, have been shown to alter the areas around and distinct from the tumor and to participate in angiogenesis, metastasis, and immune suppression. Inhibiting Angiogenesis is a viable method for treating cancer; therefore, understanding its mechanisms may lead to the identification of new drug targets. For instance, by cutting off the blood supply to cancer cells, exosomal proteins that promote angiogenesis may prevent tumor growth and spread. Exosomes have reportedly been shown to direct distant organs towards a premetastatic niche, a favorable microenvironment that encourages the survival and growth of tumor cells 42. Overall, the importance of developing cutting-edge cancer therapy strategies is emphasized by the discovery of exosomal proteins and their roles in angiogenesis.

### Importance of Understanding Exosome Protein Contents for Cancer Treatment

The exosome proteome provides important insights into the molecular pathways that underlie tumor growth and microenvironmental remodeling, supporting ongoing investigations into EVs as potential therapeutic tools. However, claims that exosome-based systems have the potential to revolutionize cancer treatment are not yet supported by sufficient clinical evidence and should be presented with greater caution. Although EVs are being explored as alternatives to conventional drug delivery platforms such as liposomes and polymeric nanoparticles 9. It may offer theoretical advantages, including improved biocompatibility and intrinsic targeting capacity; substantial translational barriers remain. These include challenges in large-scale manufacturing, standardized isolation and purification, cargo loading efficiency, storage stability, batch-to-batch reproducibility, biodistribution control, and regulatory approval pathways. Early studies once considered microvesicles (MVs) as cellular debris 78. A unique process could result in human cells producing fragments of a circular plasma membrane, according to 79, which also showed that these fragments could contain functional membrane enzymes in amounts comparable to those in the parent cells' membranes. However, the vesicles/exosomes released by different cell types into the microenvironment have not yet been clearly characterized. Two distinct methods have been used to characterize vesicle release from the cells. The endosomal membrane compartment may be the source of exosomes, which are released as MVs from the cell surface of activated cells 80. In both in vitro and in vivo settings, shedding vesicles and exosomes are present within the vesicle population. These vesicles are collectively referred to as microvesicles (MVs). Released MVs can travel through bodily fluids to distant regions or remain close to their site of release in the extracellular space. The presence of MVs in urine, plasma, milk, and CSF may be explained in part by this. Endothelial cells and other blood cells make up a smaller proportion of MVs in circulation than platelets 81. Platelet-derived MVs are also produced as microparticles; they are also called ectosomes and are made by polymorphonuclear leukocytes. Table 3 shows an exosome drug delivery system for cancer.

### Clinical Implications

Exosomes derived from natural killer cells can destroy malignancies in several ways. First, a range of cleavage particles (granzyme, perforin, and granulocyte fusion) found in exosomes generated from natural killer cells cause target cell death 93. The cytokines, chemokines, and growth factors (GM-CSF) found in natural killer cell-derived exosomes can interact with macrophages and dendritic cells to elicit immune responses 94. Natural killer cell-derived exosomes include a range of therapeutic compounds, including miR-1249-3p, and can also promote antibody-dependent cell-mediated cytotoxicity (ADCC), which facilitates the direct action of killer cells on target cells 95.

## Challenges and Future Directions

One of the most critical challenges in exosome research is the absence of universally accepted standard protocols for isolation, purification, and characterization. Multiple techniques are currently used, including ultracentrifugation, size-exclusion chromatography, precipitation-based kits, and immunoaffinity capture. However, these methods vary in yield, purity, scalability, and reproducibility. Differences in centrifugation speeds, filtration steps, and storage conditions can substantially influence exosome composition and downstream analyses. A major obstacle in biomarker validation is the absence of universally standardized protocols for exosome isolation, characterization, and quantification. Variations in ultracentrifugation parameters, precipitation methods, size-exclusion chromatography, and immunoaffinity approaches significantly affect vesicle yield and purity. Such inconsistencies complicate cross-study comparisons and contribute to irreproducible biomarker findings 96. High-profile studies demonstrate the potential of exosomal signatures, yet large multicenter prospective validation studies remain scarce. Another study emphasizes that rigorous analytical and clinical validation is essential before regulatory approval and clinical translation 76.

Although ultracentrifugation is frequently employed in the field, it has several disadvantages, including the co-isolation of non-exosomal contaminants, limited repeatability, low RNA yield, potential exosome destruction, and low sample throughput, which make it incompatible with clinical application 97. Analyses of exosomes using proteomic or flow cytometry yielded comparable findings, indicating that co-isolated plasma proteins frequently "contaminate" the separated exosome fractions and that the degree of contamination varies with the isolation technique 98.

The "contamination" issue may be resolved by directly immunocapturing exosomes from bodily fluids or cell culture supernatants; nevertheless, immunological capture relies on the detection antibody's precise binding to an antigen on the exosome surface. Therefore, it is likely that "contaminants" will hinder immune capture 99. Because high-abundance plasma proteins can contaminate exosomes and obscure low-abundance exosome-specific components, mass spectrometry may produce artifactual results 100.

Exosome research has advanced substantially; however, multiple scientific and translational barriers must be addressed before EVs can be reliably implemented as clinical diagnostic or therapeutic tools in oncology. A major limitation remains the lack of universally standardized protocols for isolation and characterization, despite recommendations from the International Society for Extracellular Vesicles in MISEV2018 and MISEV2023, which emphasize reproducibility, purity assessment, and orthogonal validation strategies. Beyond methodological variability, biomarker development remains insufficiently supported by robust quantitative validation; many proposed exosomal protein or RNA biomarkers are reported from small discovery cohorts without large independent validation sets, standardized normalization strategies, or clinically meaningful performance metrics such as sensitivity, specificity, and area under the receiver operating characteristic curve (AUC). Few studies report AUC values exceeding thresholds typically required for clinical translation (>0.80-0.85), and even fewer demonstrate added predictive value over existing diagnostic standards. Furthermore, the precise mechanisms by which exosomes modulate tumor proliferation, angiogenesis, immune remodeling, and metastatic niche formation remain incompletely defined, limiting rational therapeutic targeting. Although exosomes are proposed as natural nanocarriers for drug, RNA, and gene delivery, practical challenges persist, including scalable manufacturing, cargo-loading efficiency, targeting specificity, controlled-release kinetics, storage stability, biodistribution profiling, immunogenicity assessment, and batch-to-batch reproducibility. Importantly, few exosome-based platforms have progressed to late-phase (Phase II/III) clinical trials, underscoring the gap between preclinical promise and clinical validation. Regulatory harmonization, ethical considerations, and establishment of clinical-grade Good Manufacturing Practice (GMP) standards are also essential. Therefore, while ongoing interdisciplinary collaboration and technological innovation may expand the translational potential of exosomes, a cautious and evidence-based approach—grounded in quantitative biomarker validation and rigorous clinical testing—is required before they can meaningfully transform cancer diagnosis or therapy.

## Conclusion

Exosomes are tiny membrane-bound vesicles that can play a significant role in cancer development and in intercellular communication. Exosomal proteins have a wide range of functions and can regulate cancer cell proliferation, invasion into other tissues, and immune evasion. Understanding the functional roles of exosomal proteins in cancer is crucial for developing innovative therapeutics and improving patient outcomes. Our ability to identify and quantify exosomal proteins has significantly advanced through improved analytical techniques for profiling their protein content. These developments are crucial for deepening our understanding of exosome biology and will play a vital role in the development of effective cancer-targeted therapies. Exosomal protein targeting and improvements in exosome-based drug delivery are exciting research fields with the potential to completely change cancer treatment. To sum up, research into exosomes and the proteins that make them is an exciting and rapidly expanding field with the potential to dramatically expand our understanding of cancer biology and revolutionize the way cancer is treated.

## Generative AI statement

During the preparation of this manuscript, the authors used ChatGPT-5.0 to polish the language, improve scientific soundness, and support language editing and scientific refinement. The authors reviewed and edited the output as needed and take full responsibility for the content of this publication.

## Figures and Tables

**Table 1:** Comparison of analytical methods for exosome characterization.

Technique name	Sensitivity and Specificity	Advantage	Disadvantage	References
Mass spectrometry	High	Precise identification and quantification	High cost, Difficult sample preparation	19
Western blotting	High	Cost-effective, Relatively simple	Non-specific binding of protein, False positive results	20
ELISA	High	Measurement and quantification of specific exosomal proteins, Cost-effective.	Cross reactivity of antibodies, Sensitivity can be affected by the quantity of antibody used	21
FACS	High	Relatively fast and efficient, Sort particles based on multiple parameters simultaneously	Non-consistent results, expensive equipment	22, 23

**Table 2:** Heat shock protein's role in chemotherapy resistance

Name	Cancer type	Findings	References
HSP27	Squamous cell carcinoma of the tongue	NF-Id3 signal hyperactivation and suppression of mitochondrial caspase signal to induce multidrug resistance HSP27 knockdown and antibody therapy to reduce chemo resistance	47
	Ovarian cancer	By activating the AKT pathway and inhibiting p21, cisplatin resistance is induced.	48
	Laryngeal cancer cell	Cisplatin and staurosporin chemoresistance is induced by slowing cell development and altering actin polymerization.	49
	Pancreatic cancer	Gemcitabine chemo resistance is induced via Snail and ERCC1 activation and E-cadherin downregulation.	50
	Lung cancer	The Knockdown of HSP27 impairs TGF-β-mediated cisplatin resistance, reduces cell viability, and increases cell death.	51
	Ovarian cancer	Drugs like Paclitaxel, topotecan, and cisplatin are inducing multidrug resistance.	52
HSP40	Renal cell carcinoma	Docetaxel chemo resistance induced by DnaJB8	53
	Malignant pediatric brain tumor	DnaJD deactivation, chemotherapeutic resistance, and a possible involvement in pathogenesis	54
HSP60	Ovarian and bladder cancer	Oxaliplatin and cisplatin-induced chemoresistance induction	55
	Colorectal cancer	5-FU medication sensitivity improvement through HSP60 inhibition	55

**Table 3:** Exosomes used as drug delivery systems

	Cargo Type	Origin of Exosomes	Disease Type	Isolation or Purification Method	Drug Loading Method	Outcome	Reference
Proteins	Signal regulatory protein α	Human embryonic kidney293T cells	Cancer	Centrifugation	Transfection	Enhanced tumor cell phagocytosis	82
	Survivin-T34A	Melanoma cell lines	Pancreatic cancer	Centrifugation	NA	Apoptotic cell death	83
	Ant epidermal growth factor receptor	Mouse neuroblastoma	Epidermoid carcinoma	Ultrafiltration/size exclusion liquid chromatography	NA	Target particularity	84
	20S proteasome	Mesenchymal stem cells	Mouse myocardium	Tangential flow filtration	NA	Decreased myocardial infraction	85
Genetic substances	miRNA	Glioblastoma cells	Glioblastoma tumor	Differential centrifugation	Transfection	The provision of diagnostic data	86
	miRNA	Human cord blood endothelial colony-forming cells	Ischemic kidney injury	Centrifugation	Transfection	Decreased kidney damage and preserved kidney function	87
	Spherical nucleic acids	PC-3 cells	Prostate cancer	Centrifugation	Naturally encased	3000-fold-enhanced knockdown of miR-21	88
	siRNA	Human embryonic kidney cells (HEK293)	Breast cancer	Sequential centrifugation	Electroporation	TPD52 gene expression was downregulated up to 70% compared with non-targeted exosomes	89)
Small molecules	Paclitaxel	Prostate cancer cell lines (PC-3 and LNCaP)	Autologous prostate cancer	Differential centrifugation	Co-incubation	Increased cytotoxicity of drugs for cancer cells	90
	Doxorubicin	Immature mouse dendritic cells transfected with the vector-expressing iRGD-Lamp2b fusion proteins	Breast cancer	Centrifugation and ultrafiltration	Electroporation	Drug administration with specificity to the tumor site and tumor growth inhibition	91
	Curcumin	Tumor cells (GL26-Luc, BV2, 3T3L1, 4T1, CT26, A20, and EL-4)	Brain tumor and autoimmune encephalitis	Sucrose gradient centrifugation	Direct mixing	Reduced inflammation in the brain and slower growth of brain tumors	(D. Sun *et al*., 2010))
	Dopamine	Kunming mouse blood	Parkinson's disease	Ultracentrifugation	Co-incubation	Enhanced therapeutic effect as a result of drug delivery to the brain specifically	92
